# A Novel Branched DNA-Based Flowcytometric Method for Single-Cell Characterization of Gene Therapy Products and Expression of Therapeutic Genes

**DOI:** 10.3389/fimmu.2020.607991

**Published:** 2021-01-28

**Authors:** Laura Garcia-Perez, Marja C.J.A. van Eggermond, Elisa Maietta, Marie-Louise P. van der Hoorn, Karin Pike-Overzet, Frank J. T. Staal

**Affiliations:** ^1^ Department of Immunology, Leiden University Medical Center (LUMC), Leiden, Netherlands; ^2^ Department of Obstetrics, Leiden University Medical Center (LUMC), Leiden, Netherlands

**Keywords:** hematopoietic stem and progenitor cell, transgene, branched DNA, vector copy number, quantitative polymerase chain reaction, gene therapy, single cell, flow cytometry

## Abstract

Many preclinical and clinical studies of hematopoietic stem cell-based gene therapy (GT) are based on the use of lentiviruses as the vector of choice. Assessment of the vector titer and transduction efficiency of the cell product is critical for these studies. Efficacy and safety of the modified cell product are commonly determined by assessing the vector copy number (VCN) using qPCR. However, this optimized and well-established method in the GT field is based on bulk population averages, which can lead to misinterpretation of the actual VCN per transduced cell. Therefore, we introduce here a single cell-based method that allows to unmask cellular heterogeneity in the GT product, even when antibodies are not available. We use Invitrogen’s flow cytometry-based PrimeFlow™ RNA Assay with customized probes to determine transduction efficiency of transgenes of interest, promoter strength, and the cellular heterogeneity of murine and human stem cells. The assay has good specificity and sensitivity to detect the transgenes, as shown by the high correlations between PrimeFlow™-positive cells and the VCN. Differences in promoter strengths can readily be detected by differences in percentages and fluorescence intensity. Hence, we show a customizable method that allows to determine the number of transduced cells and the actual VCN per transduced cell in a GT product. The assay is suitable for all therapeutic genes for which antibodies are not available or too cumbersome for routine flow cytometry. The method also allows co-staining of surface markers to analyze differential transduction efficiencies in subpopulations of target cells.

## Introduction

Stem cell-based gene therapy is a promising area of medicine that is rapidly expanding with regards to clinical trials and marketing authorization. Although allogeneic hematopoietic stem cell (HSC) transplantation remains the prevailing therapeutic treatment for the correction of several types of inherited diseases, including primary immunodeficiencies, autologous genetically corrected HSC transplantation is an encouraging alternative. The first *ex vivo* gene therapy product using autologous HSC for the treatment of Adenosine Deaminase (ADA) Severe Combined Immunodeficiency (SCID) was Strimvelis (GlaxoSmithKline), and was approved in 2016 by the European Medicines Agency (EMA) (1–4). This paved the way for the clinical development of HSC-based gene therapy to treat other immunodeficiencies, including the SCIDs X-linked (5–9), Artemis (10–13), RAG1 (14), as well as X-linked chronic granulomatous disease (CGD) (15, 16) and Wiskott–Aldrich Syndrome (WAS) (17–21).

For the treatment of primary immunodeficiencies, hematopoietic stem and progenitor cells (HSPCs) are corrected *ex vivo* (reviewed by Staal et al. (22). The patient’s HSPCs are isolated, modified with the therapeutic transgene by viral transduction and the corrected cells, *i.e.* the gene therapy product, are transplanted back into the patient. Genome-integrating vectors like self-inactivating (SIN) gamma-retroviral (γ-RV) and lentiviral vectors (LV) have been used safely over the past two decades to achieve a long-lasting therapeutic effect of the transgene (23). One of the main release criteria for the treatment with a gene therapy product is to reach sufficient transgene expression, measured by the number of integrated transgene copies per target cell known as the vector copy number (VCN). The therapeutic potency of the transgene correlates positively with the proportion of transduced cells, therefore, a threshold is set for the minimal transduction efficiency required to guarantee the correction of enough cells with sufficient transgene expression for a successful and safe therapeutic outcome. However, VCN is an important parameter to control because multiple vector copies per cell can result in genotoxicity.

The golden standard technique to reliably measure VCN has been quantitative polymerase chain reaction (qPCR) of a LV sequence relative to a housekeeping gene to calculate the number of inserted vectors (24). This strategy determines the average VCN per cell in the bulk population, while only a proportion of cells carry the therapeutic vector. Therefore, the presence of non-transduced cells in the bulk population invariably underestimates the VCN of the therapeutic cells. Measuring the distribution of vector copies in corrected cells at a single-cell level is important to assess that the actual VCN is in the therapeutic range of sufficient integration without the risk of genotoxicity. Attempts to refrain from the bulk population average have been accomplished by measuring VCN in individual colony-forming cell units (CFC). Transduction efficiency determination in CFC has evolved from a green fluorescent protein (GFP) detection method (25) to more reliable and simplified qPCR assays (26, 27) that have been further validated with clinically relevant experimental data (28). Although this strategy is a step forward towards a better understanding of the cellular heterogeneity of the therapeutic product, proper single cell information is still missing. Thus, determining transgene expression with a multiparametric technology such as flow cytometry represents a quick single-cell alternative to CFC assays and an attractive alternative to bulk methods.

Here, we introduce a method based on the PrimeFlow RNA Assay^®^ (Thermo Fisher Scientific) (29, 30) generally referred to as “Branched DNA” method, as a potential new tool to characterize the gene therapy product at the single-cell level. This flow cytometry-based detection platform is inspired by fluorescent *in situ* RNA hybridization coupled with branched DNA signal amplification techniques. The technique has been adapted to single-cell suspensions and modified to detect as many as four different mRNA transcripts simultaneously at the single-cell level. Developed gene-specific oligonucleotide target probe set containing 20 to 40 probe pairs bind across the length of the mRNA. The method is a quantitative technique that preserves the cellular architecture and can provide a theoretical 8000 to 16000-fold signal amplification of the targeted mRNA to be detected by a standard flow cytometer (29–31). The branched DNA technique can be beneficial in the gene therapy field by three of its main applications. First, the technique is suitable to quantify viral RNA in infected cells, and therefore can be used to detect transduction efficiency in gene therapy products. Second, customized probes can detect target-specific RNA, *e.g.* from expanding codon-optimized therapeutic transgenes used in GT, for which available antibodies for flow cytometry are non-existent or lack sensitivity. Finally, the branched DNA technique enables heterogeneity analysis of gene expression at single-cell level, unmasking bulk cellular heterogeneity of the gene therapy product.

Taken together, we here report on adaptions of the PrimeFlow™ RNA Assay to be used in the gene therapy field as a reproducible and reliable tool to unmask the heterogeneity of the modified cell product. The percentage of transduced cells and the different promoter strengths can be analyzed, providing a unique tool to accurately assess the actual VCN per transduced cell. Furthermore, this technique shows high specificity, sensitivity and versatility, allowing it to be customized for the different therapeutic transgenes, especially when antibodies against the gene products are not available. Such valuable insights enable a more extensive characterization of gene therapy products, which helps improve the safety and therapeutic outcomes.

## Materials and Methods

### Lentiviral Vectors

Optimized *RAG1* and RAG2 sequences were synthesized by GeneArt (Regensburg, Germany) and GenScript (USA) respectively. Codon optimized RAG1 (c.o.RAG1) and codon optimized RAG2 (c.o.RAG2) were cloned into self-inactivating lentiviral pCCL plasmid resulting in pCCL-MND-c.o.RAG1 (hereafter: MND-c.o.RAG1; myeloproliferative sarcoma virus enhancer, negative control region deleted, dl587rev primer binding site substituted promoter) (32) or pCCL-EFS-c.o.RAG2 (hereafter: EFS-c.o.RAG2; elongation factor 1α short promoter) (33), pCCL-MND-c.o.RAG2 (hereafter: MND-c.o.RAG2), pCCL-PGK-c.o.RAG2 (hereafter: PGK-c.o.RAG2; human phosphoglycerate kinase-1 promoter) (34), and pCCL-UCOE-c.o.RAG2 (hereafter: UCOE-c.o.RAG2; the modified chromatin-remodeling element, devoid of unwanted splicing activity and minimized read-through activity) (35). The native RAG1 and RAG2 constructs were derived from the pRRL.PPT.PGK.GFPpre plasmid. The pRRL.PPT.SFFV.RAG1.pre (hereafter: SFFV-NativeRAG1) and the pRRL.PPT.SFFV.RAG2.pre (hereafter: SFFV-NativeRAG2) transfer vectors were constructed by replacing the PGK promoter by a MLV-derived enhancer–promoter from the spleen-focus-forming virus and the GFP sequence was replaced by human *RAG1* or *RAG2* cDNA (36). DNA sequencing of the transgene was performed to validate the gene transfer constructs. Helper plasmids pMDLg/pRRE, pRSV-Rev, and pMD2.VSVG for lentiviral production were kindly provided by L. Naldini (San Raffaele Telethon Institute for Gene Therapy, Milan, Italy) (34). Large-scale helper-plasmids were obtained from Plasmid Factory (Bielefeld, Germany).

### Vector Production

293T cells were transiently transfected with the transfer and helper plasmids using X-tremeGene HP DNA transfection reagent (Sigma-Aldrich). Lentiviruses were harvested 24, 30, and 48 h after transfection, filtered through 0.3-µm pore filters (Whatmann) and stored at −80°C. Pooled lentiviral supernatant was concentrated by ultracentrifugation (Beckman Optima™ LE-80K, rotor SW32Ti) for 16 h at 10.000 rpm and 4°C under vacuum conditions. Pellets were resuspended in StemSpan Serum-Free expansion medium (SFEM; Stemcell Technologies Inc) and aliquoted to avoid multiple freeze/thaw cycles. Since no suitable anti-RAG1 antibodies were available, we determined the viral titer using qPCR as described later on. A clinical GMP-grade MND-c.o.RAG1 vector was generated by Batavia Biosciences (Leiden, The Netherlands).The GMP-grade vector was tested and validated on murine Rag1 deficient bone marrow cells and human CD34+ cells (14).

### Human CD34+ Cell Isolation From Cord Blood (CB), Bone Marrow (BM), and Mobilized Peripheral Blood (mPB)

Human cord blood, bone marrow and peripheral blood was obtained according to the Medical Ethical Committee and IRB guidelines at Leiden University Medical Center. Cord blood mononuclear cells were separated by Ficoll (Pharmacy Leiden Academic Hospital) gradient centrifugation, frozen in fetal bovine serum (Hyclone)/10% DMSO (Sigma-Aldrich) and stored in liquid nitrogen. After thawing, human CD34^+^ cells were isolated using αCD34 MicroBead UltraPure Kit (Miltenyi Biotec). In short, cells were incubated with FcR blocking reagent and αCD34 Microbeads Ultrapure following manufacturer protocol for 30 min at 4°C. Subsequently CD34+ cells were positively selected using the appropriate ferromagnetic columns and the MACS separator (Miltenyi Biotec). CD34+ cells from BM and mPB were freshly isolated using CliniMACS (Miltenyi Biotec) by the Stem Cell lab of the Immunology department (LUMC). Hematopoietic progenitor Stem Cells (HSPC) count and purity after isolation was evaluated using a customized Flexicyte Program on NucleoCounter3000 (Chemometec). Directly isolated CD34+ cells were stimulated overnight in X-VIVO15 without Gentamycin and phenol red (Lonza) with 200 g/L Human Albumin Serum (HAS; Sanquin) or SCGM medium (CellGenix), both supplemented with 50× Pen/Strep (Gibco), 300 ng/ml human Stem Cell Factor (huSCF; Miltenyi Biotec), 100 ng/ml human Thrombopoietin (huTPO; Miltenyi Biotec), 300 ng/ml human Flt3-Ligand (huFlt3L; Miltenyi Biotec) and 10 ng/ml human Interleukin-3 (huIL3; Miltenyi Biotec).

### Murine HSPC Isolation

Lineage negative depletion was performed using the Direct Lineage Depletion kit from Miltenyi Biotec to isolate hematopoietic stem cell from frozen murine bone marrow. In short, cells were magnetically labeled with the Direct Lineage cell depletion cocktail and incubated for 10 min at 4°C. Lineage negative cells were subsequently depleted using the appropriate magnetic columns and the MACS separator (Miltenyi Biotec). Directly enriched HSPC were cultured in StemSpan (SFEM) medium supplemented with Pen/Strep (Gibco), 50 ng/ml recombinant mouse (rm) Flt3L, 100 ng/ml rmSCF and 10 ng/ml rmTPO (all from R&D Systems) at 37°C with 5%CO_2_. Depletion efficiency and purity of lineage negative population was analyzed by flow cytometry with FACSCanto (BD).

### Cell Transduction

After overnight stimulation, human CD34+ or murine lineage negative bone marrow cells were transduced in the appropriate complete medium with the different lentiviruses. Various transduction methods were used. 1] Cells were transduced using 4 µg/ml protamine sulphate (PS; Sigma-Aldrich) and with or without spin-occulation at 800*g* and 32°C for 1 h. 2] Human cells were transduced adding 100 mg/ml LentiBOOST™ (Sirion Biotech) together with the lentiviral supernatant. 3] Cells were transduced using the combination of 4 µg/ml protamine sulphate and 100 mg/ml LentiBOOST™. Cells were cultured at 37°C, 5% CO_2_ for 24 h in medium supplemented with cytokines as described above. Cells were cultured for 9 days in the appropriate culture medium for further DNA, RNA and branched DNA assay analysis.

### PrimeFlow™ RNA Assay

PrimeFlow™ RNA assay (ThermoFisher) was performed on HSPCs after 9 days in culture following the manufacturer protocol divided over 2 days. All buffers are included in the PrimeFlow™ RNA assay kit and specific target probe sets for huRPL13A (type 4), muACTB (type 4), c.o.RAG1 (type 1 and type 6), c.o.RAG2 (type 1 and type 6), native RAG1 (type 10) and Native RAG2 (type 1) were designed by and purchased from ThermoFisher. When applicable, cell surface staining was performed for 30 min at 2°C to 8°C with fluorochrome conjugated antibodies, such as CD34-PE (8G12; BD Biosciences), CD34-BV510 (581; Biolegend; AB_2563856), and CD90-BV605 (5E10; Biolegend; AB_2562281). Cells were then fixed for 30 min at 2° to 8°C. After permeabilization, cells were fixed a second time for 1 h at RT with Fixation buffer 2. A hybridization step was performed by incubating the cells with the appropriated target probe sets for 2 h at 40°C. Samples were stored over night at 2°C to 8°C in the dark. The day after, pre-amplification and amplification of the hybridization was performed by 2 consecutive incubations of 1.5 h at 40°C with the pre-Amplification mix and subsequently the Amplification mix. Finally, cells were incubated with the label probe sets for an hour at 40°C. Cells were measured by flow cytometry on FACS-CantoII and LSR Fortessa X-20 (BD Biosciences) or sorted on FACSAria II (BD Biosciences) and the data was analyzed using FlowJO software (Tree Star).

### Determination Vector Copy Number (VCN) and *Gene* Expression by RT-qPCR

After 9 days in culture (to prevent detection of pseudotransduction), qPCR was used for the quantitative analysis of genomic lentiviral RNA, proviral DNA copies and transgene mRNA expression of the transduced HSPCs using WPRE (Woodchuck Hepatitis Virus Posttranscriptional Regulatory Element), c.o.RAG1, c.o.RAG2, ABL-I, and PTBP2 (Polypyrimidine Tract Binding Protein 2) as targets (**Table S1**). Total RNA from single cell suspensions was purified using RNeasy Mini kit (Qiagen) and reverse transcribed into cDNA using Superscript III kit (Invitrogen). Genomic DNA was extracted from single cell suspensions using the GeneElute Mammalian Genomic DNA kit (Sigma-Aldrich). DNA and RNA concentration were measured by NanoDrop (ThermoFisher). VCN was determined on DNA samples by the detection of viral WPRE normalized to genomic household gene PTBP2. The levels of transgene expression were determined on cDNA samples, by normalizing the transgene to the expression of the *ABL-I* gene. qPCR was performed using TaqMan Universal Master Mix II (ThermoFisher) in combination with specific probes for indicated genes from Universal Probe Library (Roche). Primers and probes used are listed in **Table S1**. PCR reactions were performed on the StepOnePlus Real-Time PCR system (ThermoFisher). All samples were run in triplicate.

### Statistical Analysis

Statistics were calculated and graphs were generated using GraphPad Prims 8. Statistical significance was determined with two-tailed Pearson r correlation coefficients. Analyses, such as linear regression, non-linear regression, and area under the curve, have been used across the experiments.

## Results

### Branched DNA Assay: Suitable to Detect Transgene Transcription in Clinically Relevant HSPC

**Figure 1:**
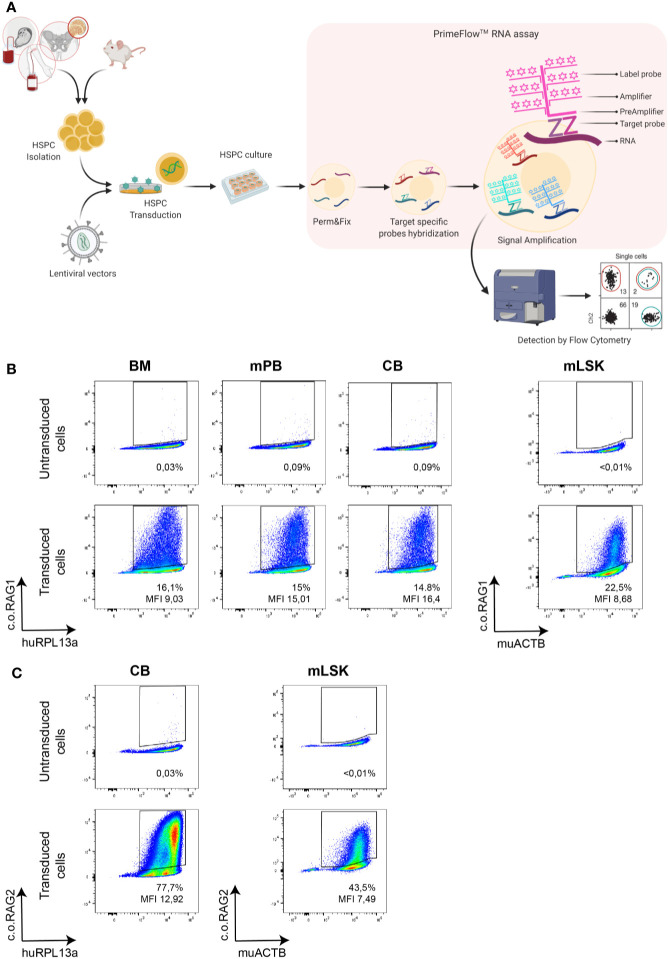
Branched DNA method is suitable to detect diverse therapeutic transgenes in cells of interest. **(A)** Schematic overview of the analytical process and the branched DNA methodology. Human and murine hematopoietic progenitor stem cells (HSPC) isolated from various sources like cord blood (CB), bone marrow (BM) or mobilized peripheral blood (mPB) were transduced with lentiviral vectors and cultured for 9 days. Cells were harvested and split for DNA isolation, RNA isolation and branched DNA assay, in which cells are first permeabilized and fixed, then target-specific probes are hybridized with the mRNA and finally signal is amplified. Cells can then be analyzed by standard flow cytometry [Created with BioRender.com]. **(B)** FACS plot showing the detection of the therapeutic c.o.RAG1 transgene of transduced human CD34+ cells (1000 viral particles/cell; MND-c.o.RAG1 LV) isolated from BM, mPB or CB as well as in murine isolated HSPC. **(C)** FACS plot confirming the detection of the therapeutic c.o.RAG2 transgene in transduced cord blood CD34+ cells and in murine HSPCs (1800 Viral particles/cell; MND-c.o.RAG2 LV). Human RPL13a and murine ACTB housekeeping mRNAs have been used as internal controls. Percentage of positive cells and MFI (ratio MFI positive/negative population) are depicted in each plot.

Gene therapy for immunodeficiencies is commonly performed by inserting a normal copy (native or codon optimized) of the defective gene into the patient’s CD34+ enriched HSPCs isolated from bone marrow (BM) or mobilized peripheral blood (mPB). The branched DNA technique was therefore tested on isolated CD34+ cells from BM, mPB and cord blood (CB). Cells were transduced with the therapeutic codon optimized (c.o.) RAG1 LV (14), cultured for 9 days (to limit the detection of early, but temporary expression from non-integrated transgenes also referred to as pseudo-transduction) and analyzed by the branched DNA assay with customized probes developed for *c.o.RAG1* mRNA and the housekeeping *RPL13a* mRNA as an internal control (See method overview in **Figure 1A**). As shown in **Figure 1B**, *RPL13a* but not *c.o.RAG1* was detected in non-transduced HSPCs. However, transduced cells from all sources revealed an evidently positive *c.o.RAG1* population. Although cells were transduced with the same number of viral particles per cell (VP/cell) and infectious genomes (MOI) (1000 VP/cell; MOI 3.6), a different transduction efficiency can be observed both by the percentage of transduced cells and the mean fluorescent intensity (MFI) with different values for CB (14.8%; 16.4 MFI),BM cells (16.1%; 9.03 MFI) and mPB cells (15%; 15.01 MFI), indicating.differences in cell permissiveness of human HSPCs depending on the source. Preclinical development of gene therapy is primarily performed in animal models like mice. The branched DNA assay was therefore tested on isolated transduced LSK cells (Lineage negative Sca1+ c-Kit+ cells). A positive *c.o.RAG1* population was clearly detected (22.5%; 8.68 MFI), while no signal was detected in non-transduced LSK cells (percentage below 0.01). For murine cells, *ACTB* was used as household internal mRNA control. Interestingly, the probe sets can be customized to any gene of interest, and consequently this assay can be adapted to a broad variety of therapeutic transgenes. For example, *c.o.RAG2* probes were also developed and validated (**Figure 1C**) on transduced CD34+ CB cells and murine LSK cells. In both cases, a well-defined positive *c.o.RAG2* transduced population was detected (77.7%; 12.92 MFI and 43.5%; 7.49 MFI respectively). Hence, the novel branched DNA technique allows the reliable detection of therapeutic transgenes in progenitor cells of human and mouse origin.

### High Specificity to Detect Transduced Cells Within the Bulk HSPC Population

**Figure 2:**
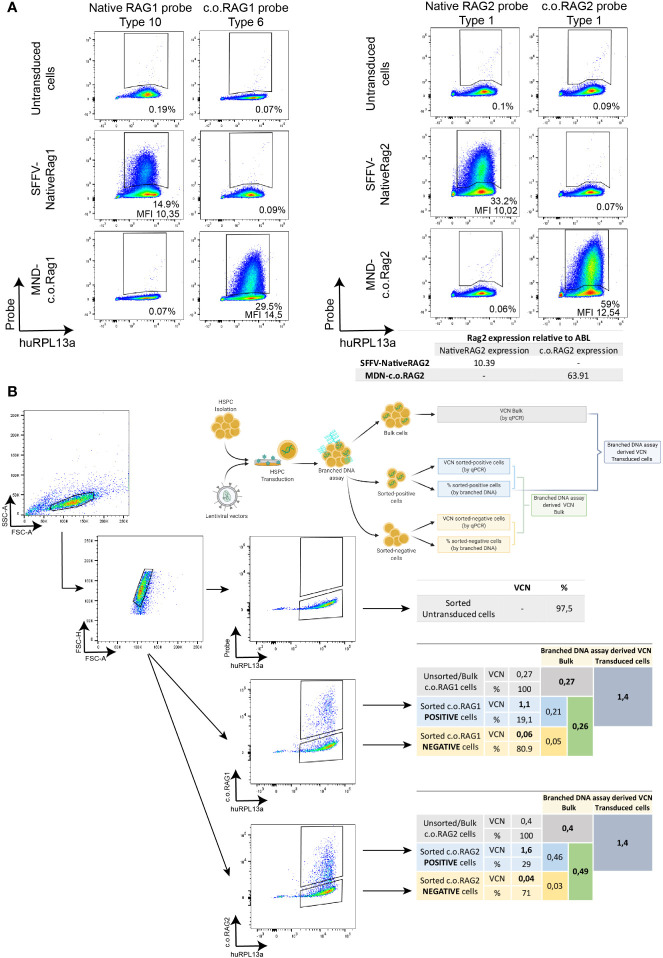
Specificity of the branched DNA technique to detect transduced cells within the bulk HSPC population. **(A)** FACS plots representing the specificity of the DNA branched method using different lentiviral vectors (SFFV-NativeRAG1, SFFV-Native RAG2, MND-c.o.RAG1, and MND-c.o.RAG2) and specific probe sets for the detection of Native RAG1, RAG2 or codon optimized RAG1 or RAG2. Percentage of positive cells and MFI (ratio MFI positive/negative population) are depicted per plot. Human RPL13a housekeeping mRNAs have been used as internal control. Table showing the detection by qPCR of RAG2 expression (Native or codon optimized) in the different transduced cells. **(B)** FACS plots depicting the sorting strategy of non-transduced, MND-c.o.RAG1 and MND-c.o.RAG2 transduced cells. C.o.RAG1 or -2 positive and negative populations were sorted after branched DNA assay, and VCN was determined by qPCR in the unsorted and sorted populations. Diagram and tables show the VCN determined by qPCR and percentages (%) measured by flow cytometry after branched DNA assay, as well as the branched DNA derived VCN of the sorted cells in the bulk population (VCN sorted population x % sorted population) and the branched DNA derived VCN of the transduced cells from the bulk VCN and the known percentage of positive cells (VCN bulk population**/**% positive population). Human RPL13a housekeeping mRNAs have been used as internal control.

A variety of LVs and probe sets were used to assess the specificity of this novel branched DNA technique. CD34+ cells enriched from CB were transduced with LVs expressing the native or codon optimized *RAG1* (SFFV-RAG1 or MND-c.oRAG1). Transduction efficiency was assessed by the branched DNA assay with specific probe sets developed against native *RAG1* and *c.o.RAG1* mRNAs (**Figure 2A**_left panel). A positive native *RAG1* population (14.9%) was only detected in transduced cells with the specific native RAG1 probe sets, but not with the c.o.RAG1 probe sets. Similarly, *c.o.RAG1* transduced cells were only detected with the c.o.RAG1 probe sets (29.5%). The same approach was used to assess specificity of the RAG2 probe sets (**Figure 2A**_right panel) where native *RAG2* expression was only detected by the native probe sets (33.2%) and *c.o.RAG2* positive cells were only identified with the c.o.RAG2 probe sets (59%). Quantitative PCR of *c.o.RAG2* or *RAG2* relative to *ABL-I* expression confirmed these results (inset below **Figure 2A**_right panel). Thus, our developed probe sets were highly specific and accurately discriminated between native sequences and codon optimized mRNAs, without cross-reactivity between similar sequences (e.g. 82% similarity between c.o.RAG1 and native RAG1 sequences and 94.5% similarity between c.o.RAG2 and native RAG2 sequences).

Another relevant parameter is to elucidate whether the positive detected cells are accurately reflecting the proportion of cells with the transgene insertion. Therefore, CD34+ cells transduced with either c.o.RAG1 or c.o.RAG2 were sorted after 9 days in culture based on positivity using the branched DNA assay. Positive and negative c.o.RAG1 or c.o.RAG2 populations were sorted (all RPL13a positive cells) and VCN was determined by qPCR in the sorted populations, as well as in the bulk population before sorting (**Figures 2B**, **S1**). Both transduced positive populations revealed high VCNs (1.1 for c.o.RAG1 and 1.6 for c.o.RAG2) whereas the negative sorted populations showed insignificant VCNs (0.06 and 0.04 respectively). Importantly, positively sorted cells present a considerably higher VCN than the VCN assessed in the bulk population by qPCR (1.1 sorted *vs.* 0.27 bulk for c.o.RAG1 and 1.6 sorted *vs.* 0.4 bulk for c.o.RAG2). With 19.1% of the bulk properly c.o.RAG1 transduced with a VCN of 1.1 and a negative population representing 80.1% (VCN of 0.06), the calculated bulk VCN obtained was 0.26; very close to the 0.27 assessed directly by qPCR in bulk cells. Similarly, calculated c.o.RAG2 VCN in the bulk (derived from sorted cell data) is 0.49 while VCN determined by qPCR in the bulk was 0.4. These data show that both the branched DNA and qPCR methods are extremely accurate. Conversely, knowing the VCN measured by qPCR in bulk cells (0.27 for c.o.RAG1 and 0.4 for c.o.RAG2) together with the fraction of actual transduced cells (19.1% c.o.RAG1 cells and 29% c.o.RAG2 cells) is sufficient to determine the VCN of the actual transduced cells. Here this was 1.4 in both cases, relatively close to the VCN determined in the positively sorted cells (1.1 and 1.6, respectively).

Altogether, the branched DNA method shows high specificity and accuracy in discriminating between close sequences and detecting positively transduced cells that express the therapeutic transgene. Moreover, this assay provides key information on transduction efficiency of the gene therapy product, allowing a reliable calculation of the real actual VCN of the portion per transduced cells.

### Proper Sensitivity to Detect Therapeutic Transgenes

**Figure 3:**
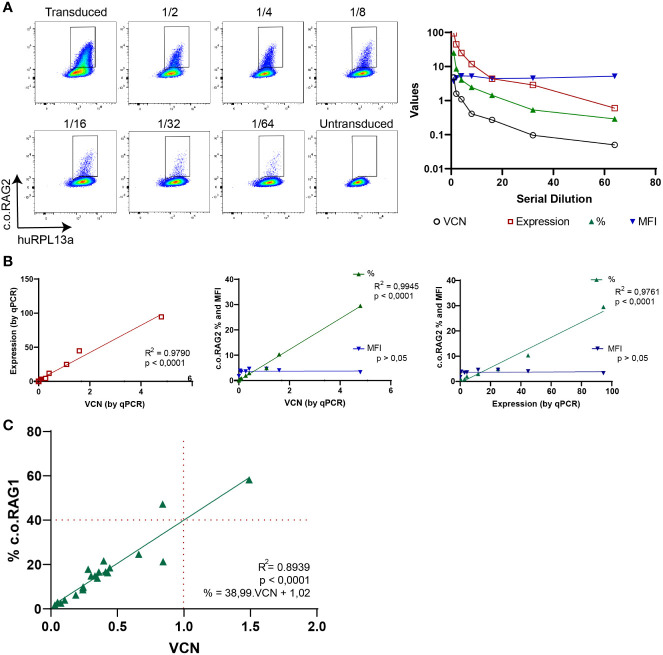
Branched DNA assay sensitivity to detect therapeutic transgenes. **(A)** FACS plots showing the positive c.o.RAG2 population of transduced CD34+ cells in a serial dilution with non-transduced cells, from 1/2 to 1/64. VCN, c.o.RAG2 expression, % and MFI measured in the serial dilution are represented in a graph. Human RPL13a housekeeping mRNAs have been used as internal control. **(B)** Correlation between different parameters have been studied: VCN (by qPCR) vs c.o.RAG2 expression (by qPCR), VCN vs % and MFI (by flow cytometry) and c.o.RAG2 expression (by qPCR) vs % and MFI. (Two-tailed, Pearson r correlation coefficients). **(C)** Correlation between VCN detected by qPCR and % or MFI determined by flow cytometry including a total of 24 independent experiments on CD34+ cells (mPB and BM) transduced with MND-c.o.RAG1 at different viral particles per cell and different transduction protocols. Red dashed line= % c.o.RAG1 cells at VCN=1. (Two-tailed, Pearson r correlation coefficients; linear regression).

To test the sensitivity of the branched DNA technique, serial dilution of transduced cells was performed and analyzed both by the branched DNA assay and qPCR (**Figure 3A**). C.o.RAG2 transduced cells were cultured for 9 days and serially diluted by mixing with non-transduced cells. While MFI values stayed constant with the serial dilution, the percentage of positive cells decreased with each dilution. The VCN and expression level measured by qPCR also decreased accordingly. A significant correlation between the standard parameters VCN and gene expression measured by qPCR (R^2^ = 0.979, p < 0.001) is found (**Figure 3B**). The percentage of positive cells as measured by the branched DNA method also reveal a high correlation with the VCN (R^2^ = 0.9945, p < 0.001) and gene expression level (R^2^ = 0.9761, p < 0.001). Finally, data from up to 23 experiments of c.o.RAG1 transduced cells, either from BM or mPB, shows high reproducibility of the novel assay (**Figure 3C**) with significant correlation between VCN and the percentage of transduced cells (R^2^ = 0.8939, p < 0.001). As seen in **Figure 3C**, an average of 40% of cells are properly transduced in mPB or BM with our MND-c.o.RAG1 LV when a VCN of 1 was detected by qPCR in the bulk population, resulting in an actual VCN of 2.5 in the transgene-positive cells (40% of the gene therapy product). This can vary depending on the cell source and the vector used. When c.o.RAG2 LV was used to transduce CD34+ cells isolated from CB, on average 31% of the population was transduced when a VCN of 1 was detected, increasing to a VCN of 3.2 in the transduced cells (**Figure S2**). As modified cells only represent a portion of the bulk HSPCs, the VCN of the modified, potentially therapeutic cells is higher than the determined by qPCR (see **Table 1**) and the transgene insertion is generally underestimated by bulk measurements.

**Table 1:** Real VCN.

Cells	Transgene	Bulk VCN	% transduced cells	Real VCN of transduced cells
mPB	c.o.RAG1	0.03	0.97	2.89
mPB	c.o.RAG1	0.08	2.55	2.94
mPB	c.o.RAG1	0.19	6.23	3.00
mPB	c.o.RAG1	0.24	9.92	2.45
mPB	c.o.RAG1	0.35	13.80	2.53
mPB	c.o.RAG1	0.84	21.30	3.96
mPB	c.o.RAG1	0.40	21.60	1.84
mPB	c.o.RAG1	0.33	14.90	2.23
mPB	c.o.RAG1	0.33	14.80	2.24
mPB	c.o.RAG1	0.28	17.80	1.57
mPB	c.o.RAG1	0.84	47.20	1.78
mPB	c.o.RAG1	1.49	58.20	2.56
mPB	c.o.RAG1	0.66	24.60	2.68
BM	c.o.RAG1	0.43	16.30	2.62
BM	c.o.RAG1	0.43	16.30	2.62
BM	c.o.RAG1	0.36	16.60	2.17
BM	c.o.RAG1	0.05	2.95	1.73
BM	c.o.RAG1	0.44	18.50	2.39
BM	c.o.RAG1	0.41	16.70	2.47
BM	c.o.RAG1	0.03	1.78	1.69
BM	c.o.RAG1	0.24	8.63	2.78
BM	c.o.RAG1	0.24	9.55	2.51
CB	c.o.RAG2	1.35	59.00	2.29
CB	c.o.RAG2	1.15	29.30	3.94
CB	c.o.RAG2	2.10	62.50	3.36
CB	c.o.RAG2	1.86	31.30	5.94
CB	c.o.RAG2	1.74	46.70	3.73
CB	c.o.RAG2	0.02	1.17	1.71
CB	c.o.RAG2	0.11	6.53	1.61
CB	c.o.RAG2	1.06	28.00	3.79
CB	c.o.RAG2	2.52	51.20	4.92
CB	c.o.RAG2	2.04	70.30	2.90
CB	c.o.RAG2	0.38	29.00	1.31

Collectively, this data indicate that the results of the branched DNA technique correlate to those of qPCR methods and constitutes a highly sensitive and reproducible method to measure the percentage of transduced cells.

### Branched DNA Assay as a Novel Gene Therapy Tool

**Figure 4:**
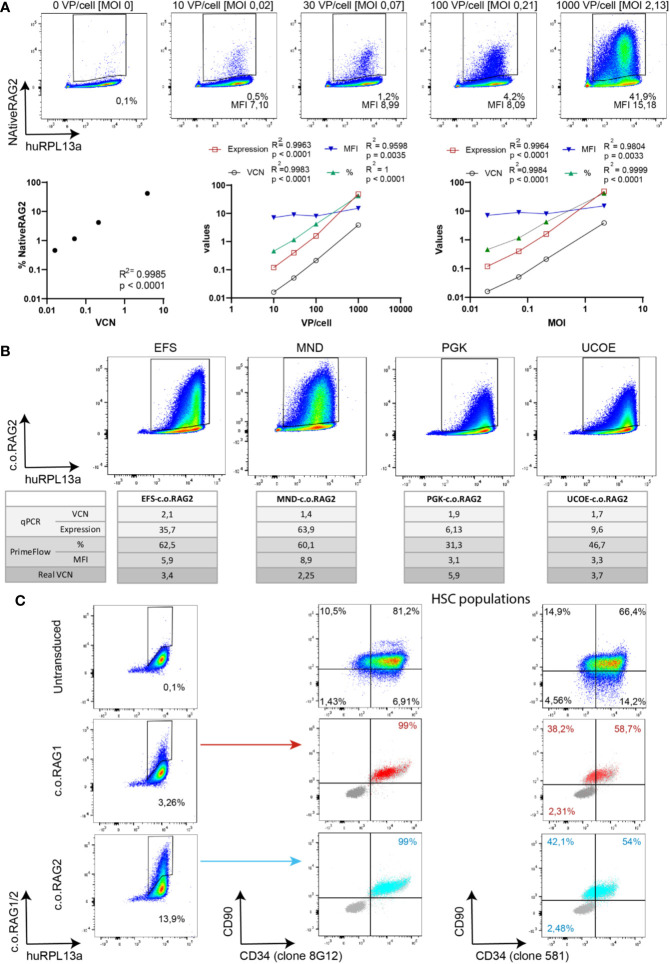
Branched DNA method as a novel gene therapy tool. **(A)** Branched DNA technique as a virus titration tool: Representative FACS plots of the detected positive NativeRAG2 population in CD34+ cells with increasing viral concentration. % and MFI depicted in each case. Human RPL13a housekeeping mRNAs have been used as internal control. Graphs represent correlation between VCN vs % and Viral Particles/cell or MOI vs VCN (by qPCR), expression (by qPCR), % (by branched DNA assay) and MFI (by branched DNA assay). (Two-tailed, Pearson r correlation coefficients). **(B)** Branched DNA assay for promoter strength visualization: FACS plots representing the c.o.RAG2 population detected by flow cytometry after transduction of CD34+ cells at similar VCN with different constructs including different promoters (EFS-c.o.RAG2, MND-c.o.RAG2, PGK-c.o.RAG2, and UCOE-c.o.RAG2). Human RPL13a housekeeping mRNAs have been used as internal control. VCN, c.o.RAG2 expression, % and MFI results are depicted in the table. **“**Real**”** VCN is calculated in each case as VCN (by qPCR)**/**% (by flow cytometry). **(C)** FACS plots showing c.o.RAG1 or c.o.RAG2 transduced cells determined after 4 days in culture. Transduced cells co-expressed CD34 and CD90 markers (dark grey = unstained control, light grey = non-transduced control, blue=c.o.RAG2 positive cells, red=c.o.RAG1 positive cells). HSC population FACS plots represent the bulk population. Human RPL13a housekeeping mRNAs have been used as internal control.

Determining the virus titer is important to calculate the quantity of virus needed to achieve efficient and reproducible transduction of primary cells. Enriched CD34+ cells from CB were transduced with increasing amounts of native RAG2 LV (SFFV-Native RAG2) and analyzed by qPCR and branched DNA technique after 9 days in culture (**Figure 4A**). An increasing percentage of positive native RAG2 cells was detected by branched DNA-flow cytometry (0.5%, 1.2%, 4.2%, and 41.9%) with increasing numbers of VP/cell and MOI (10 VP/cell [0.02 MOI]; 30 VP/cell [0.07 MOI]; 100 VP/cell [0.21 MOI] and 1000 VP/cell [2.13 MOI] respectively). Just as in **Figure 3B**, there was a high and significant correlation (R^2^ = 0.9985, p < 0.0001) between the VCN determined by qPCR and the percentage of positive cells detected by the branched DNA technique. In addition, all parameters measured by qPCR (VCN and expression) and the branched DNA assay (% and MFI) significantly correlated with the VP/cell and MOI with R^2^ values higher than 0.9804 and p < 0.0001 (**Figure 4A**, graphs).

Therefore, both the standard qPCR and the branched DNA assay correlate with the viral titration. Interestingly, the percentage of transduced cells can reach a plateau at a lower VP/cell and MOI) than the VCN determined in bulk (**Figures S3A, B**). Overall VCN kept increasing with rising viral particles but not the proportion of modified cells, implying a potential increase of insertions within the same cell population.

Identifying differences in promoter strengths could reveal different gene expression intensity patterns. Isolated HSPCs were transduced with various c.o.RAG2 LVs carrying different promoters (EFS (33), MND (32), PGK (34) and UCOE (35), see Material & Methods section). After 9 days in culture, transduced cells were split and analyzed by qPCR and branched DNA technique (**Figure 4B**). Bulk VCN detected in the different transductions was measured as 1.76 ± 0.27. Although similar VCN were determined along the conditions, a wide range of c.o.RAG2 gene expression was detected by qPCR ranging from 6.13 with the PGK promoter to 63.9 with the strong MND promoter. Similarly, different percentages and MFIs were detected by the branched DNA assay, depending on the promoter used (from 31.3% and 3.1 MFI with PGK to 60.1% and 8.9 MFI with MND promoter). Even though bulk VCN were similar across the conditions, the calculated VCN per transduced cells differs across promoters because the actual percentage of transduced cells highly differ from 60% to 31%. Taken together, the branched DNA method accurately reflects the MFI of transduced cells that together with the percentage gives an indication of the strength of the promoter used.

The last benefit of the branched DNA technique is that it can be combined with the staining of other cell markers, allowing to more thoroughly study transduction of HSPCs sub-populations. Transduced cells stained for *c.o.RAG1* or *c.o.RAG2* using the branched DNA method were co-stained with CD34 and CD90 antibodies to define CD34^+^CD90^+^ HSCs in the bulk CD34-enriched HSPC population (**Figure 4C**). Positive c.o.RAG1 and c.o.RAG2 cells were detected 4 days after transduction (3.26% c.o.RAG1 and 13.9% c.o.RAG2 positive populations). Importantly, cells adequately expressing c.o.RAG1 or c.o.RAG2, co-expressed CD34 and CD90 (CD34 staining with two different clones) visualizing the transduction of HSCs within a CD34-enriched HSPC population with our therapeutic transgenes by flow cytometry.

The single cell-based data make the branched DNA technique a valuable tool to be used in determining the optimal virus titration, to compare different constructs and to depict transduction of sub-population defined by extra markers. Notably, it is the first time to depict single cell information directly in clinically applicable therapeutic plasmids. Most importantly, determination of the VCN per effectively transduced cell represents a determinant factor to define the composition of a heterogeneous gene therapy product.

## Discussion

We herein describe and validate a branched DNA method coupled with flow cytometry to directly measure mRNA and thus assess transduction efficiency and transgene expression at the single-cell level. As an additional parameter, the MFI indicates the expression level of the therapeutic gene on a per cell basis. Until now, most of our assumptions of transgene expression are based upon the VCN as a bulk average that changes with a different transduction efficiency. Using the branched DNA assay, we accurately evaluate the proportion and VCN of the therapeutic cells present in the heterogeneous bulk CD34+ product, particularly with a relevant c.o.RAG1 vector (14). In fact, the lack of information regarding expression levels in individual cells became a problem in our initial experiments aimed at developing RAG1 gene therapy (37). It was unclear based on bulk Q-PCR data whether there was a modest level of transduction in all target cells, or if a small subset was preferentially targeted with much higher efficiencies. Our later studies indicated a significant heterogeneity in transduction levels, even when using an envelope protein (VSV-G) that should transduce all cells (14). Thus, using this assay, large numbers of events can be reliably collected by standard flow cytometry, generating sufficient statistical power which is especially important when analyzing rare events.

This branched DNA assay proves to be suitable to detect transgene expression like *c.o.RAG1* in potentially therapeutic CD34+ enriched cells from numerous sources like BM, mPB or CB, as well as in the murine HSPCs used in most of the pre-clinical studies. Enriched CD34+ cells from different sources have different cell composition, proliferation index and lifespan (38–42), resulting in diverse transduction permeability as reflected by the different percentages of gene marking efficiencies when transduced with the same amount of virus and infectious genomes. With its customized probe sets, the branched DNA technique is versatile because it can be adjusted to detect a broad variety of clinically applicable therapeutic genes. The developed probe sets are highly specific, discriminating between similar sequences of the native and the codon-optimized mRNA of a gene without cross-reactivity. Moreover, this method allows an accurate detection of the modified cells invariably containing and expressing the therapeutic transgene versus the unmodified cells. The correlation between the detection of transduction with the standard qPCR (VCN) and novel method (%) is significant and reproducible over independent experiments and constructs. However, the reliable detection of transduced cells using the branched DNA method shows better sensitivity than the standard qPCR method in detecting small portions of positive cells. While the branched DNA technique is a robust assay to study transduction efficiency by means of percentage of transduced cells, the mean fluorescent intensity (MFI) is more variable as it depends on the quality of the lasers in each instrument. To standardize MFI values, the ratio between the positive population MFI and the negative population was calculated. In addition, intensity of the expressed transgene reflected by the MFI also depends on the expression level at the harvesting time (days in culture, cell proliferation), adding an extra variable within the MFI parameter. While MFI is stable for the same promoter, it can of course differ for different promoters and can be used to determine relative promoter strength. Finally, the branched DNA method is an attractive alternative when proper antibodies against the transgene product are not available. Although the protein level is not measured directly, a single-cell flow cytometry readout of mRNA transcribed from the introduced transgene was previously not available. Co-staining with other cellular markers can provide more specific information on target sub-populations. Ideally, a complete antibody panel including all CD34+ sub-populations can reveal single-cell information on the specific targeted subpopulation and their differences with the bulk CD34+ cells. Notably, this novel method to detect single cell information directly in clinically applicable therapeutic cells eliminates the need for GFP carrying constructs. However, the successful use of cell surface co-staining requires individual optimization as antibody epitopes and fluorochromes are not always compatible with the permeabilization/fixation protocol. Altogether, the branched DNA assay shows high specificity, sensitivity and reproducibility to reliably determine transduction efficiency of HSPCs by measuring the precise frequency of transduced cells at a single cell level. Previously unavailable single-cell data is unmasking the heterogeneity of the gene therapy cell product.

Knowing the suitable specificity, sensitivity and reproducibility; the branched DNA assay can be used as a tool to analyze virus titration in primary cells, compare promoter strength of different constructs, to identify the transduced HSPCs sub-populations by co-staining with extra markers. The novel single-cell data supports a reliable re-interpretation of the actual VCN within the modified therapeutic cells as opposed to the average in the bulk population becoming a determinant factor to measure the composition of the gene therapy product and to evaluate the risk of genotoxicity of the clinical vectors. The higher actual VCN on modified cells may indicate an increased risk of genotoxicity on the transduced cells which could be underestimated when relying on bulk measurements. Correspondingly, the branched DNA technique revealed an underestimation of the actual VCN in the potentially therapeutic cells by the presence of a rather sizable fraction of non-transduced cells in the bulk CD34+ product population. Indeed, around 40% of the bulk HSPCs are modified with the clinical c.o.RAG1 vector when aiming at an average of one insertion per cell (VCN=1); a lower portion than the 63% expected from a mathematical approach (Poisson distribution) generated by Fehse et al. (43). Therefore, the VCN of transduced cells has been underestimated, indicating an actual 2.5 times higher VCN in the therapeutic cells than what has been assessed by qPCR in the bulk. Assessment of this correction factor of VCN in therapeutic cells can vary between vectors (research vs GMP grade or transgene) like for our research grade c.o.RAG2 LV with a lower percentage of targeted cells (30%) than c.o.RAG1 LV. Independent calculation of this factor per clinically applicable vector might therefore be advisable.

In the perspective of clinical gene therapy, the branched DNA method is intended to be implemented as an extra assay to collect additional safety data and better characterization of the gene therapy product and the potentially therapeutic cells. The murine model for X-linked SCID (IL2rg) suggests that the threshold of functional corrected HSPCs required to reconstitute immune function is around 10% of the total transplanted cells (44); although additional studies with the branched DNA assay are needed to assess this with a clinically relevant vector. A more accurate analysis of transduction efficiency and the number of potentially therapeutic cells to be transplanted can be correlated to the immune reconstitution and transplantation outcome. This additional criterion for transduction efficiency would reveal the minimum number of transduced, therapeutic cells with a safe “real” VCN with a successful transplantation outcome. The increased usage of codon optimized transgenes in the new versions of SIN LV that are being developed allows a successful customization of the probe sets for the different therapies. This novel strategy could be implemented for ongoing clinical trials for immunodeficiencies like X-linked SCID (5–9), ADA SCID (45), Artemis SCID (10–13), X-linked chronic granulomatous disease (CGD) (15, 16, 46) or WAS (17–21, 47), as well as for other diseases with a similar gene therapy approaches like Pyruvate Kinase deficiency (48), Fanconi Anemia (49) or hemoglobinopathies (50–52).

To conclude, we introduce a novel branched DNA technique as an additional tool to accurately assess transduction efficiency at a single cell level and to measure VCN of transduced cells in gene therapy for immunodeficiencies and other modalities, revealing underestimated VCN and heterogeneity of the gene therapy product.

## Data Availability Statement

The original contributions presented in the study are included in the article/**Supplementary Material**. Further inquiries can be directed to the corresponding author.

## Ethics Statement

The studies involving human participants were reviewed and approved by LUMC. The patients/participants provided their written informed consent to participate in this study. The animal study was reviewed and approved by LUMC.

## Author Contributions

LG-P, MCJAE, and EM performed experiments and data analysis. FJTS, KP-O, and LG-P designed experiments and the overall study concept. M-LH provided clinical relevant resources. LG-P, KP-O, and FJTS wrote the manuscript. All authors contributed to the article and approved the submitted version.

## Funding

This work was supported in part by ZonMW E-RARE grant (40-419000-98-020) and received funding from the European Union’s Horizon 2020 research and innovation program under grant agreements No. 666908 (SCIDNET) and No. 755170 (RECOMB).

## Conflict of Interest

The authors declare that the research was conducted in the absence of any commercial or financial relationships that could be construed as a potential conflict of interest.
