# Supplementary material for: A Novel Branched DNA-Based Flowcytometric Method for Single-Cell Characterization of Gene Therapy Products and Expression of Therapeutic Genes
Source: Front Immunol. 2021 Jan 28;11:607991. doi: 10.3389/fimmu.2020.607991 (PMC7876092; doi:10.3389/fimmu.2020.607991)
Supplement: Supplementary file 1 [file DataSheet_1.docx]

# Supplementary Figures:

## Figure S.1: Specificity of the branched DNA assay to detect transduced cells (duplicate sorting)

FACS plots depicting the sorting strategy of non-transduced, MND-c.o.RAG1 and MND-c.o.RAG2 transduced cells. C.o.RAG1 or 2 positive and negative populations were sorted after branched DNA assay, and VCN was determined by qPCR in the unsorted and sorted populations. Diagram and tables show the VCN determined by qPCR and percentages (%) measured by flow cytometry after branched DNA assay, as well as the branched DNA derived VCN of the sorted cells in the bulk population (VCN sorted population x % sorted population) and the branched DNA derived VCN of the transduced cells from the bulk VCN and the known percentage of positive cells (VCN bulk population / % positive population). Human RPL13a housekeeping mRNAs have been used as internal control.

## Figure S.2: Expected portion of transduced cells when VCN of 1 is obtained by qPCR

Correlation VCN determined by qPCR and percentage of transduced cells detected by branched DNA technique. A total of 11 independent experiments of transduced CD34+ cells isolated from cord blood and transduced with various c.o.RAG2 LVs at different multiplicity of infection. Red dashed line= % c.o.RAG2 cells at VCN=1. (Two-tailed, Pearson r correlation coefficients; linear regression).

## Figure S.3: Proportion of transduced cells reached plateau earlier than bulk VCN

A) Branched DNA technique as a titration tool: Representative FACS plots of the detected positive c.o.RAG2 population in CD34+ cells with increasing viral concentration. Human RPL13a housekeeping mRNAs have been used as internal control. B) Graphs represent correlation between VCN and % vs Viral Particles/cell (VP/cell) or MOI. (Non-linear fit regression).
