# Supplementary figures and images for: A Novel Branched DNA-Based Flowcytometric Method for Single-Cell Characterization of Gene Therapy Products and Expression of Therapeutic Genes
Source: Front Immunol. 2021 Jan 28;11:607991. doi: 10.3389/fimmu.2020.607991 (PMC7876092; doi:10.3389/fimmu.2020.607991)

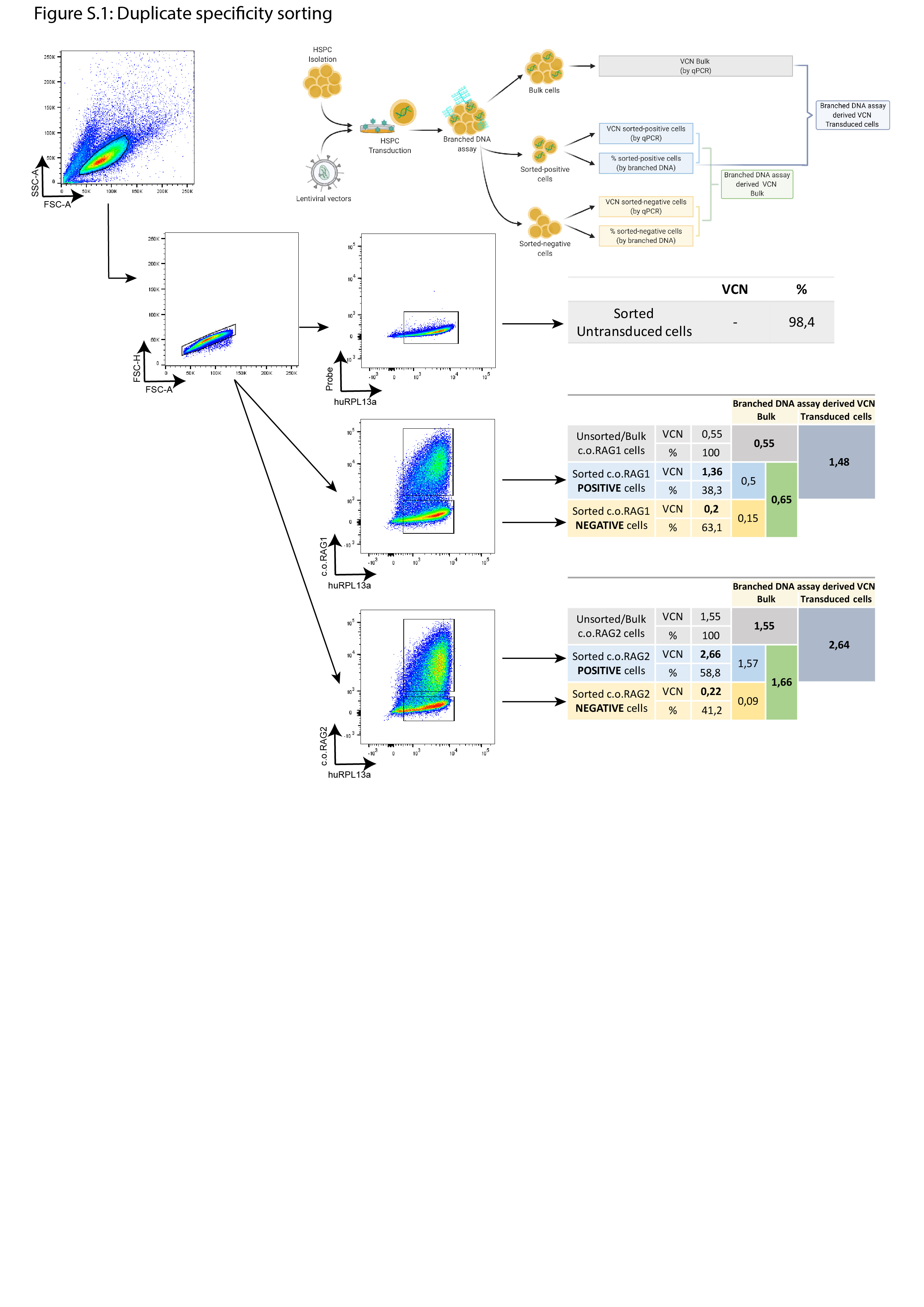

Supplement: Supplementary file 2 [file Image_1.jpg]

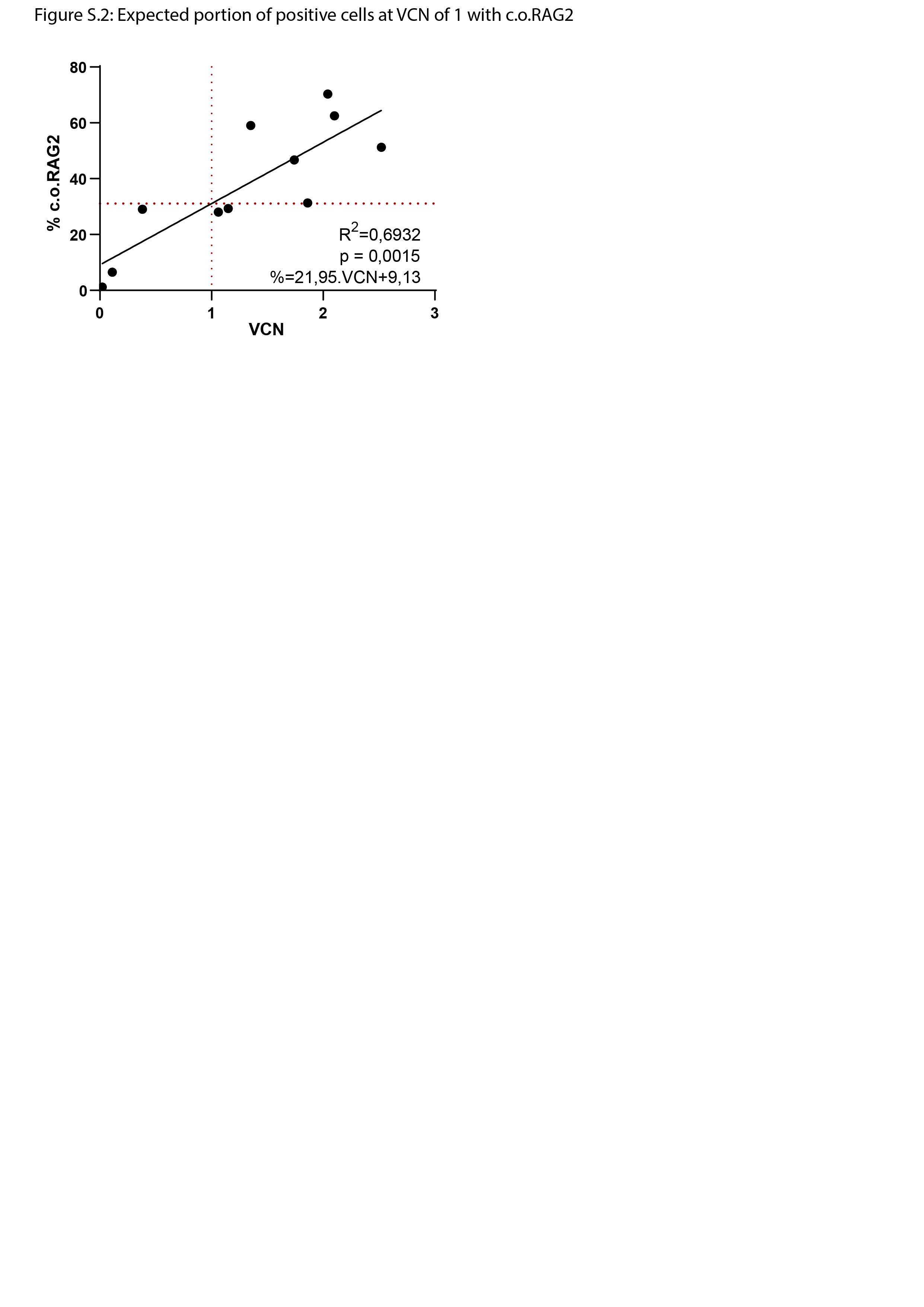

Supplement: Supplementary file 3 [file Image_2.jpg]

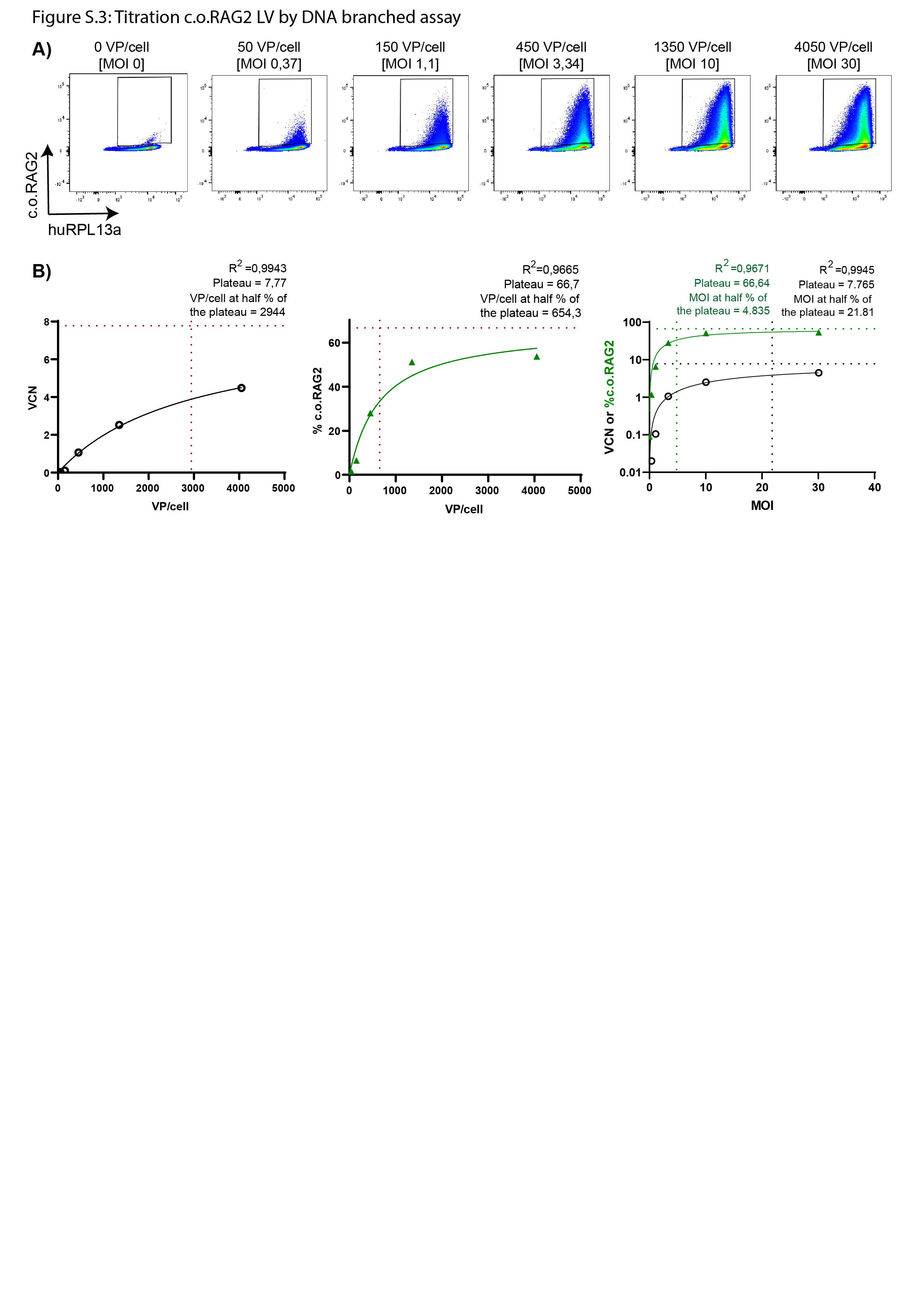

Supplement: Supplementary file 4 [file Image_3.jpg]
