# Supplementary material for: A Novel Branched DNA-Based Flowcytometric Method for Single-Cell Characterization of Gene Therapy Products and Expression of Therapeutic Genes
Source: Front Immunol. 2021 Jan 28;11:607991. doi: 10.3389/fimmu.2020.607991 (PMC7876092; doi:10.3389/fimmu.2020.607991)
Supplement: Supplementary file 5 [file Table_1.pdf]

Table S1
List of primers and probes

Determination VCN and transgene expression

Description	Orientation	DNA sequence 5'-3'
ABL1	FW	5’-TGGAGATAACACTCTAAGCATAACTAAAGGT-3’
	RV	5’-GATGTAGTTGCTTGGGACCCA-3’
	Probe	5’FAM-CCATTTTTGGTTTGGGCTTCACACCATT- TAMRA 3’
c.o.Rag1	FW	5' CAACTGCAAGCACGTGTTCTG 3'
	RV	5' GCAGTAGCTGCCCATCACTTT 3'
	Probe	5'FAM AGAGTGTGCATCCTGCGGTGCCT TAMRA 3'
c.o.RAG2	FW	5’-TCTGAAACCGGGTATTGGAT- 3’
	RV	5’-GGCACCCATGTATTAATGTCC-3’
	Probe	Probe: 56 probe library Roche, FAM NFQ
NativeRAG2	FW	5-TGGATGTAAAGCATAACCATGTCA-’ 3’
	RV	5’-GGGAGGTAGCAGGAATCCTTAGA-3’
	Probe	[6FAM]-TCCTGCTACCTCCCTC-TAMRA
PTBP2	FW	5’-TCTCCATTCCCTATGTTCATGC-3’
	RV	5’-GTTCCCGCAGAATGGTGAGGTG-3’
	Probe	[JOE]-ATGTTCCTCGGACCAACTTG-[BHQ1]
WPRE	FW	5’- GAGGAGTTGTGGCCCGTTGT-3’
	RV	5’-TGACAGGTGGTGGCAATGCC-3’
	Probe	[6FAM]-CTGTGTTTGCTGACGCAAC-[BHQ1]
